# Beneficial effects of preoperative oral nutrition supplements on postoperative outcomes in geriatric hip fracture patients: A PRISMA-compliant systematic review and meta-analysis of randomized controlled studies

**DOI:** 10.1097/MD.0000000000027755

**Published:** 2021-11-24

**Authors:** Wen-Yi Lai, Yu-Chi Chiu, Kuo-Ching Lu, I-Tao Huang, Pei-Shan Tsai, Chun-Jen Huang

**Affiliations:** a Department of Anesthesiology, Wan Fang Hospital, Taipei Medical University, Taipei, Taiwan; b Integrative Research Center for Critical Care, Wan Fang Hospital, Taipei Medical University, Taipei, Taiwan; c Emergency Department, Redcliffe Hospital, QLD, Australia; d School of Public Health, Faculty of Medicine, University of Queensland, QLD, Australia; e Department of Nursing, Wan Fang Hospital, Taipei Medical University, Taipei, Taiwan; f Research Center of Big Data and Meta-analysis, Wan Fang Hospital, Taipei Medical University, Taipei, Taiwan; g School of Nursing, College of Nursing, Taipei Medical University, Taipei, Taiwan; h Department of Anesthesiology, School of Medicine, Taipei Medical University, Taipei, Taiwan; i Graduate Institute of Clinical Medicine, College of Medicine, Taipei Medical University, Taipei, Taiwan.

**Keywords:** complication, elderly, hip fracture, length of hospital stay, mortality, oral nutrition

## Abstract

**Background:**

Geriatric hip fracture patients often present malnutrition during admission, which leads to higher morbidity and mortality. Protein-based oral nutrition supplements may improve nutritional status. We conducted this systematic review and meta-analysis of randomized controlled trials (RCTs) according to the PRISMA guidelines to elucidate whether preoperative nutrition supplements can improve postoperative outcomes in geriatric hip fracture patients.

**Methods:**

Only RCTs conducted to compare postoperative outcomes between geriatric hip fracture patients (>60 years old) receiving preoperative oral protein-based nutrition supplement (ONS group) and those who receiving regular diet (Control group) were included. PubMed, Embase, and Cochrane Central Register of Controlled Trials were searched from inception until August, 2021. Postoperative outcomes, including complications, length of hospital stay, and in-hospital mortality, were assessed.

**Results:**

A total of 5 RCTs with 654 geriatric hip fracture patients (ONS group: 320 subjects; Control group 334 subjects) were included. Our data revealed that postoperative complications risk in the ONS group was significantly lower than in the Control group (odd's ratio: 0.48, 95% confidence intervals [CI]: 0.26–0.89, *P* = .02, *I*^2^ = 64%). However, no significant differences in the length of hospital stay (standardized mean difference: −0.35 days, 95% CI: −1.68 to 0.98 days, *P* = .61, *I*^2^ = 0%) and the risk of having postoperative in-hospital mortality (odd's ratio: 1.07, 95% CI: 0.43–2.63, *P* = .89, *I*^2^ = 54%) between these 2 groups were observed. Quality assessment revealed high risk of bias and significant data heterogeneity (*I*^*2*^>50%) in most included RCTs.

**Conclusion:**

Preoperative protein-based oral nutrition supplements exert beneficial, but limited, effects on postoperative outcomes in geriatric patients with hip fracture undergoing surgery.

## 1 Introduction

Geriatric patients are often presented malnutrition, mainly due to smaller than required energy intake.^[1,2]^ Moreover, hip fracture may cause inflammation, hypermetabolism, muscle mass loss, weight loss, and subsequently worsen malnutrition status in geriatric patients.^[3]^ Clinical data showed that geriatric hip fracture patients with malnutrition are associated with an increased risk of having postoperative complications during hospitalization, causing longer length of hospital stay and higher mortality.^[4–7]^ Clinical data further showed that perioperative nutrition supplements can shorten hospitalization duration of geriatric hip fracture patients.^[8]^ Several clinical studies also demonstrated that geriatric hip fracture patients receiving perioperative nutrition supplement tend to have lower incidences of postoperative complications than those who receiving regular diets.^[9–11]^

Notably, comparing with intravenous or nasogastric tube routes, oral nutrition supplements have been shown with the best cost-effectiveness in improving nutritional status of geriatric hip fracture patients.^[8]^ Clinical data further highlighted that protein is responsible for the beneficial effects of perioperative nutrition supplements.^[12]^ In line with this notion, we conjectured that perioperative protein-based oral nutrition supplements may exert beneficial effects on improving postoperative outcomes in geriatric hip fracture patients. Data from previous systematic review and meta-analysis studies partially supported this concept, as meta-analysis of data from relevant randomized controlled trials (RCTs) confirmed that perioperative protein-based oral nutrition supplements could exert beneficial effects in improving postoperative complications.^[13–15]^ However, perioperative protein-based oral nutrition supplements exerted no significant effects in improving postoperative mortality.^[13,14]^

Because adverse impacts of hip fracture on geriatric patients occur immediately after the incidence,^[3]^ therefore the best timing for starting administration of nutrition supplements theoretically should be immediately upon hospitalization rather than after surgery. The outcomes reported in the previous systematic review and meta-analysis studies^[13–15]^ were extrapolated from literatures of postoperative oral nutrition supplements. To date, meta-analysis data regarding the impacts of preoperatively administered protein-based oral nutrition supplements on postoperative outcomes in geriatric hip fracture patients remains lacking.

To elucidate further on this issue, we conducted this systematic review and meta-analysis of published clinical studies. Our hypothesis was that geriatric hip fracture patients receiving preoperative protein-based oral nutrition supplements had better postoperative outcomes than those who receiving regular diets.

## 2 Materials and methods

### 2.1 Ethics, data source, and search

This systematic review and meta-analysis study was exempt from ethical approval, according to the principles of the Institutional Review Board of Taipei Medical University, Taipei, Taiwan. This systematic review and meta-analysis was conducted according to the detailed guideline of the Preferred Reporting Items for Systematic Reviews and Meta-analysis (PRISMA) statement.^[16]^ We searched relevant articles in databases of PubMed, Embase, and the Cochrane Central Register of Controlled Trials from inception until August, 2021. The combination of searched terms were “oral nutrition” or “oral supplement” or “nutrition” or “protein,” “hip surgery” or “hip fracture” or “femoral neck fracture” or “intertrochanteric fracture” or “subtrochanteric fracture,” “old” or “elderly” or “aged,” “postoperative complications” or “mortality” or “length of hospitals stay.” Patients more than 60 years old of age were defined as elderly patients. We also browsed the reference lists for potentially eligible studies.

### 2.2 Study selection and outcomes

Two authors (W-YL and Y-CC) independently screened the studies and extracted data on the relevant outcomes. According to the title and abstract, only RCTs investigating patients aged over 60 years who had hip fractures (femoral neck, intertrochanteric or subtrochanteric, acetabulum fractures) and underwent surgery (open reduction and internal fixation or arthroplasty) with intervention of preoperative protein-based oral nutrition supplements (orally taking protein or high-protein diets) were included. Notably, studies including patients with multiple fractures or pathological fractures, nutritional support after discharge, and participants of hip fracture without surgical treatment were excluded. The study outcomes were serum albumin levels, the overall postoperative complication rates, length of hospital stay, and mortality during the hospitalization period.

### 2.3 Quality assessment and data extraction

The quality of method of each included RCT was rated by 2 authors (W-YL and Y-CC) independently using the Cochrane Handbook for Systemic Review of Intervention 5.1.0. In addition, we developed a data extraction sheet and extracted the data from each included study. Information extracted of the included studies: study design; age of participants; intervention measurements; postoperative outcomes. For resolving disagreement relating to an article's quality assessment, the 2 authors (W-YL and Y-CC) would discuss with each other or consult with a senior researcher (P-ST or C-JH) to reach a final decision.

### 2.4 Data synthesis and analysis

Perioperative data regarding patients, intervention, and outcomes as described above were extracted from the included studies. We used Review Manager 5.3 software to analyze the data and adopted a 95% confidence interval. For the measurement outcomes, the standardized mean difference was calculated. For the enumeration outcomes, the odds ratio was calculated. An *I*^*2*^ value of 50% or lower represented “no observed heterogeneity,” whereas an *I*^*2*^ value of more than 50% indicated “heterogeneity” among the included studies.

## 3 Results

### 3.1 Study identification

**Figure 1:**
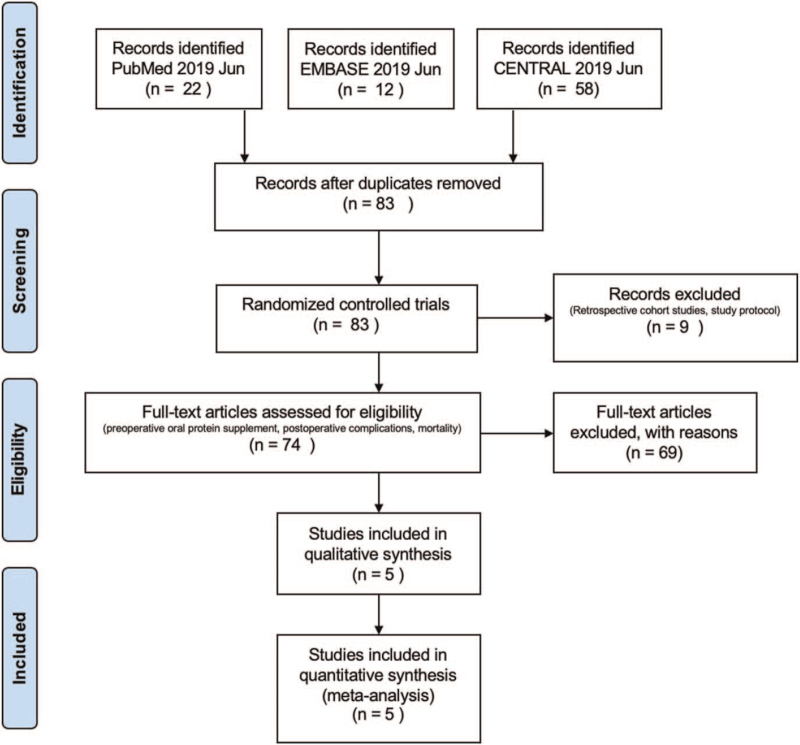
Study identification algorithm, established according to the Preferred Reporting Items for Systematic Reviews and Meta-Analyses statement.

Figure 1 illustrated study identification algorithm. A total of 92 studies were initially identified in the searched databases. Among them, 9 duplicates were removed. The remaining 83 studies were further screened. Studies lacking original data, retrospective studies, oral nutritional supplement given after the surgery were excluded. As a result, a total of 5 RCTs^[12,17–20]^ comprising of 654 geriatric hip fracture patients, including 320 subjects receiving preoperative protein-based oral nutritional supplement (the ONS group, n = 320) and 334 subjects receiving regular diets (the Control group, n = 334), were included for systematic review and meta-analysis.

### 3.2 Details of included RCTs

Details of the included RCTs were listed in Table 1. All subjects in the included RCTs^[12,17–20]^ were geriatric patients (≥ 60 years old of age) with hip fracture. All subjects in the ONS group received preoperative high protein-based oral nutrition supplements, started upon hospitalization prior to surgery, and lasted throughout the duration of hospitalization. Of note, Duncan et al,^[18]^ Tkatch et al, ^[19]^ and Delmi et al^[20]^ provided oral nutrition supplements prepared by the nutritionists from the hospital, whereas Espaulella et al^[12]^ and Botella-Carretero et al^[17]^ provided oral nutrition supplements using commercialized high-protein based supplements.

**Table 1:** Characteristic of included studies.

Trial, year	Number of participants	Fracture type	Description	Participant age (years)	Intervention	Control
Delmi et al, 1990 ^[20]^	59	Femoral neck fracture	Randomized controlled trial	82	Oral protein supplement	Standard hospital diet
Tkatch et al, 1992 ^[19]^	62	Proximal femur fracture	Randomized controlled trial	ONS: 83.2 ± 1.3, Control: 81.3 ± 1.6	Oral protein supplement with vitamin and minerals	No protein supplement with vitamin and minerals
Espaulella et al, 2000 ^[12]^	171	Hip fracture	Double-blinded randomized, placebo controlled trial	ONS: 82.4 ± 6.6, Control: 82.7 ± 7.3	Oral protein and antioxidant supplement by Clinical Nutrition SA, Spain	Placebo
Duncan et al, 2006 ^[18]^	302	Hip fracture	Open prospective randomized controlled trial	ONS: 81.5 ± 0.9 Control: 80.5 ± 1.3	Oral protein and energy supplement by dietetic assistants	Standard hospital diet
Botella-Carretero et al, 2010 ^[17]^	60	Hip fracture	Randomized, controlled, open, paralleled two-arms clinical trial	ONS: 85.1 ± 4.4, Control: 82.1 ± 7.3	Oral energy protein supplements by Fortimel	Standard hospital diet

Data are mean ± standard deviations. ONS: subjects receiving protein-based oral nutrition supplements. Control: subjects receiving regular diets.

### 3.3 Quality assessment

**Figure 2:**
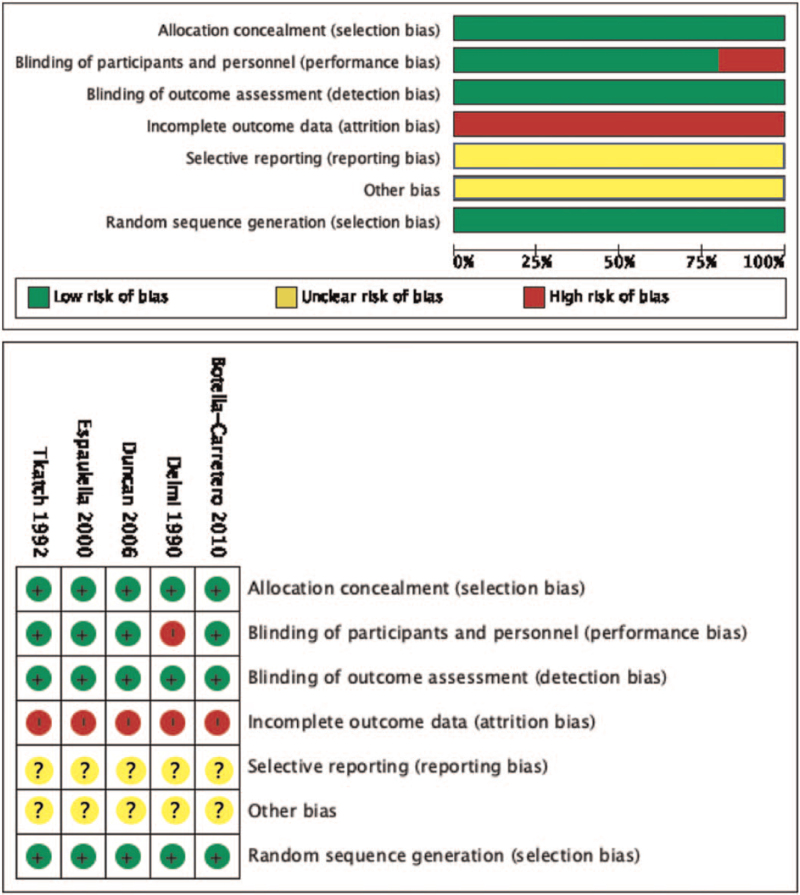
Quality assessment of the included studies.

Figure 2 illustrates the data of quality assessment of the included RCTs. All included RCTs had randomized the patients by computer programming, indicating low allocation bias of all studies. Two included RCTs^[18,20]^ had high performance bias, as no placebo was given to the Control group and the nurses dispensing the preoperative oral nutrition supplements were not blinded to the intervention. Most of the included RCTs^[17–20]^ had high attrition bias, because they failed to report either postoperative complications or mortality data. All of the included RCTs had unclear reporting bias due to unblinding of the data extraction participants whom may had selectively reported the functional ability and postoperative complications of the patients. Collectively, these data indicated low grade evidence level of the included RCTs.

### 3.4 Impacts of preoperative oral nutritional supplements on postoperative outcomes

Details of postoperative outcomes of the included RCTs were listed in Table 2.

**Table 2:** Postoperative outcomes.

Trial, year	Number of participants	Serum albumin (gm/dL)	Postoperative complications	Hospital stays (days)	Mortality (numbers (%))
Delmi et al, 1990 ^[20]^	59	Preoperative: ONS: 3.7 ± 0.6, control: 3.6 ± 0.6 Postoperative: ONS: 3.4 ± 0.3, Control: 2.7 ± 0.4	Bedsore, severe anemia, infection, gastrointestinal ulcer	—	ONS: 4 (14%) Control: 3 (9%)
Tkatch et al, 1992 ^[19]^	62	—	Infection, bed sore, cardiac failure, anemia, digestive disturbance	ONS: 23.5 ± 7.85 Control: 24.7 ± 7.85	ONS: 3 (9%) Control: 2 (6%)
Espaulella et al, 2000 ^[12]^	171	—	Delirium, urinary infection, bed sore	ONS: 16.4 ± 6.6 Control: 17.2 ± 7.7	ONS: 17 (21%) Control: 10 (12%)
Duncan et al, 2006 ^[18]^	302	—	Overall complications (no details described)	ONS: 16 ± 18 Control: 32 ± 49	ONS: 6 (6%) Control: 16 (10%)
Botella-Carretero et al, 2010 ^[17]^	60	Preoperative: ONS: 3.2 ± 0.4 Control: 3.4 ± 0.4 Postoperative: ONS: 2.9 ± 0.3, Control: 2.5 ± 0.4	Vomiting, Diarrhea, infection, cognitive impairment	ONS: 13.3 ± 4.3 Control: 12.8 ± 4.0	—

Data are mean ± standard deviations. ONS: subjects receiving protein-based oral nutrition supplements. Control: subjects receiving regular diets.

#### 3.4.1 Serum albumin

Two included RCTs reported data of serum albumin levels.^[17,20]^ Botella-Corretero et al^[17]^ reported preoperative serum albumin levels that measured before surgery and postoperative serum albumin levels that measured at 48 hours after surgery. Data from Botella-Corretero et al^[17]^ revealed that serum albumin levels significantly decreased after surgery in both the Control group (preoperative vs postoperative: 3.4 ± 0.4  vs 2.5 ± 0.4 gm/dL) and the ONS group (3.2 ± 0.4 vs 2.9 ± 0.3 gm/dL) (both *P* < .001). Of note, the postoperative serum albumin levels in the ONS group were significantly higher than in the Control group (2.9 ± 0.3 vs 2.5 ± 0.4 gm/dL; *P* = .002). Delmi et al^[20]^ also reported preoperative (measured before surgery) and postoperative (measured at 14 days after surgery) serum albumin levels. Data that significant decreases in serum albumin levels after surgery in both the Control group (preoperative vs postoperative: 3.6 ± 0.6 vs 2.7 ± 0.4 gm/dL) and the ONS group (3.7 ± 0.6 vs 3.4 ± 0.3 gm/dL), and that the postoperative serum albumin levels in the ONS group were significantly higher than in the Control group (3.4 ± 0.3 vs 2.7 ± 0.4 gm/dL) (all *P* < .05) were also reported by Delmi et al.^[20]^

#### 3.4.2 Postoperative complications

**Figure 3:**
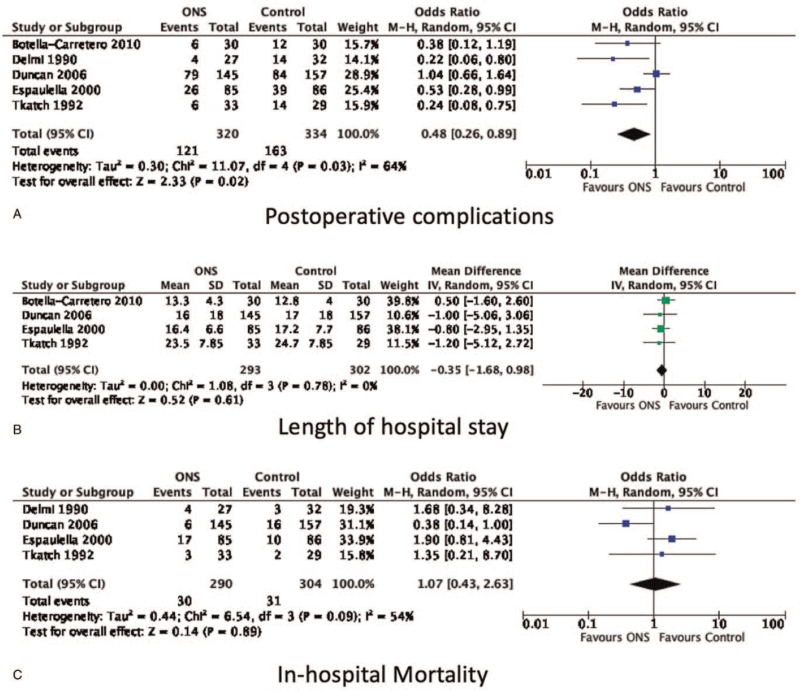
Forest plots illustrating postoperative outcomes, including (A) risk of postoperative complications, (B) length of hospital stay (days), and (C) risk of in-hospital mortality in the ONS group versus the Control group. ONS: subjects receiving preoperative protein-based oral nutrition supplements. Control: subjects receiving regular diets. CI: confidence intervals.

Five included RCTs reported the data of overall postoperative complication rates^[12,17–20]^. Espaulella et al^[12]^ reported a lower overall postoperative complication rate in the ONS group than in the Control group (31% vs 45%). The investigated complications included delirium, urinary tract infection and bed sore. Botella-Carretero et al^[17]^ also reported a lower overall postoperative complication rate in the ONS group than in the Control group (20% vs 40%) and the investigated complications included vomiting, diarrhea, infection, and cognitive impairment. Duncan et al^[18]^ reported that the overall postoperative complication rates in the ONS and the Control groups were comparable (54% vs 53%), but without providing the details of investigated complications. Tkatch et al^[19]^ reported a lower overall postoperative complication rate in the ONS group than in the Control group (18% vs 48%) and the investigated complications included infection, bed sore, cardiac failure, anemia, and digestive disturbance. A lower overall postoperative complication rate in the ONS group comparing to that in the Control group (15% vs 44%) was also reported by Delmi et al^[20]^ and the investigated complications included bed sore, severe anemia, infection, and gastrointestinal ulcer. Of note, our meta-analysis data revealed that the risk of having postoperative complications was significantly lower in the ONS group (n = 320) than in the Control group (n = 334) (random effect OR: 0.48, 95% Cl: 0.26–0.89, *P* = .02, *I*^2^ = 64%; Fig. 3A). Significant data heterogeneity was observed.

#### 3.4.3 Length of hospital stay

The length of hospital stay was the duration of hospitalization in the orthopedic ward since the day of admission. Hospitalization in the rehabilitation ward or the second hospital was not included. Four included RCTs reported the data of length of hospital stay^[12,17–19]^. Espaulella et al^[12]^ (16.4 days in the ONS group and 17.2 days in the Control group), Duncan et al^[18]^ (16 days in the ONS group and 17 days in the Control group), Tkatch et al^[19]^ (23.5 days in the ONS group and 24.7 in the Control group) all reported shorter length of hospital stays in the ONS group than in the Control group. In contrast, Botella-Carretero et al^[17]^ (13.3 days in the ONS group and 12.8 days in the Control group) reported longer length of hospital stays in the ONS group than in the Control group. Our meta-analysis data showed that the length of hospital stay in the ONS group (n = 293) and the Control group (n = 302) were not significantly different (random effect standardized mean difference: −0.35 days, 95% confidence intervals: −1.68 to 0.98 days, *P* = .52, *I*^2^ = 0%; Fig. 3B). No data heterogeneity was observed.

#### 3.4.4 In-hospital mortality

Four included RCTs reported the data of in-hospital mortality after surgery.^[12,17–19]^ Espaulella et al^[12]^ (21% in the ONS group and 12% in the Control group), Tkatch et al^[19]^ (9% in the ONS group and 6% in the Control group) and Delmi et al^[20]^ (14% in the ONS group and 9% in the Control group) all reported higher in-hospital mortality rates in the ONS group than in the Control group. In contrast, Duncan et al^[18]^ reported a lower in-hospital mortality rate in the ONS group than in the Control group (6% vs 10%). Our meta-analysis data revealed that the risk of having in-hospital mortality after surgery in the ONS group (n = 290) and the Control group (n = 304) were not significantly different (random effect OR: 1.07, 95% Cl: 0.43–2.63, *P* = .89, *I*^2^ = 54%, Fig. 3C). Again, significant data heterogeneity was observed.

## 4 Discussion

It is well established that nutritional status is an important influencing factor for postoperative complications and mortality in geriatric patients undergoing surgery.^[10,11,21]^ This concept is confirmed by previous data that malnutrition increases the risks of having postoperative complications, having longer length of hospital stay and having in-hospital mortality in geriatric hip fracture patients.^[4–7]^ Because protein-based oral nutrition supplements might exert beneficial effects on improving nutritional status of elderly hip fracture patients,^[8]^ we thus hypothesized that geriatric hip fracture patients receiving preoperative protein-based oral nutrition supplements may have better postoperative outcomes.

This hypothesis is partially confirmed by our data, as the meta-analysis data demonstrated that preoperative protein-based oral nutrition supplements could decrease the risk of having postoperative complications (up to an approximate 50% decrease) in geriatric hip fracture patients undergoing surgery. Though the trends of having a shorter length of hospital stay (−0.35 days) in the ONS group were noted when comparing to that in the Control group; whereas, our meta-analysis data revealed that the difference in this postoperative outcome between the ONS and the Control groups did not reach statistical significance. The difference in the risk of having in-hospital mortality after surgery between the ONS and the Control groups was not significantly different, either. These data demonstrated that preoperative protein-based oral nutrition supplements did not exert significant effects on the length of hospital stay and the risk of having in-hospital mortality after surgery in geriatric patients with hip fracture. Collectively, these data provide clear evidence to demonstrate for the first time that preoperative protein-based oral nutrition supplements could exert beneficial, but limited, effects on improving postoperative outcomes in geriatric hip fracture patients.

Nutritional status is an important influencing factor for postoperative complications and mortality^[10,11,21]^. As above mentioned, inflammation and hypermetabolism caused by hip fracture may worsen the malnutrition status in geriatric patients.^[1,2]^ Serum albumin level is highly associated with nutritional status and has long been used as a marker for nutritional status.^[6,22–24]^ Hübner et al^[25]^ reported that early drop of postoperative serum albumin may reflect the magnitude of surgical trauma, and is correlated to clinical outcomes. Of note, data from the included RCTs^[17,20]^ revealed significant decreases in serum albumin levels after surgery in subject receiving regular diet. These data highlighted that the processes of hospitalization and surgery, in conjunction with the insult of hip fracture, can significantly decrease serum albumin levels in geriatric hip fracture patients. Data from the included RCT^[17]^ further revealed that the decreases in serum albumin levels after surgery can also be observed in geriatric hip fractures patients receiving preoperative oral nutrition supplements, but with a smaller magnitude of decrease when comparing to those who receiving regular diets. These data demonstrated that preoperative protein-based oral nutrition supplements can mitigate, but cannot reverse, the trend of decreases in serum albumin levels after surgery in geriatric hip fracture patients. Clinical data indicated that geriatric patients with hypoalbuminemia may be associated with a higher prevalence of sepsis, longer hospital stays, and higher prevalence of readmission and mortality after surgery.^[6,22–24]^ In line with this notion, it is thus reasonable to observe our data that preoperative protein-based oral nutrition supplements could exert only limited beneficial effects on improving postoperative outcomes in geriatric hip fracture patients. Based on our data, we believe that efforts more than preoperative protein-based oral nutrition supplements are needed if better postoperative outcomes, especially mortality, in geriatric hip fracture patients are intended.

Certain study limitations do exist. First, this study investigated the effects of preoperative protein-based oral nutrition supplements on improving postoperative outcomes in geriatric hip fracture patients. However, the included RCTs employed different formulations of protein-based oral nutrition supplements, either prepared by the nutritionists from the hospital^[18–20]^ or using commercialized high-protein-based supplements.^[12,17]^ These supplement formulations varied in the contents, especially the amount of protein. In addition, subject's daily intake of protein-based oral nutrition supplements was not strictly controlled in all the included RCTs. As a result, one would expect inconsistency in daily protein supplement intake among subjects in the ONS group. Moreover, the included RCTs also varied in the formulations of control. Two included RCTs^[12,19]^ employed placebo control and 3 included RCTs^[17,18,20]^ employed regular hospital diet as the control. Similarly, one would expect inconsistency in daily nutrition intake among subjects in the Control group. These inconsistencies will thus lower the grade of evidence level of this study. Second, 4 included RCTs^[12,17,19,20]^ reported gender data of subjects and 1 included RCT^[18]^ did not. The majority subjects included in both the ONS group (female/male: 139/32) and the Control group (female/male: 147/34) were female elderly, according to the data reported in 4 included RCTs.^[12,17,19,20]^ Therefore, the results reported in this meta-analysis study were mainly derived from female elderly. This factor will also lower the grade of evidence level of this study. Moreover, prefracture health, nutritional, and functional status are strong predictors for postoperative outcomes in elderly hip fracture patients.^[26]^ However, only 1 included RCT^[12]^ reported subject's preoperative functional and cognitive status. Detailed information regarding subjects’ prefacture health status, especially chronic disease (i.e., diabetes mellitus, obesity, hypertension, anemia, kidney disease, depression, etc), was not reported in most of the included RCTs. Information regarding subjects’ prefracture nutritional status and habitual diet was not reported in most of the included RCTs, either. Lacking of the above-mentioned crucial information will also lower the grade of evidence level of this study. Third, the protein-based oral nutrition supplements investigated in this study consisted only of basic protein ingredients. Therefore, the impacts of some enriched supplements, for example, with immune nutrition, calcium β-hydroxy-β-methylbutyrate, and/or vitamin D,^[9,27]^ in this regard remain un-studied. Fourth, the ratio of C-reactive protein/albumin can be a predictor of 28-day mortality in critically ill patients.^[28]^ However, the 2 included RTCs^[17,20]^ that measured albumin levels did not measure C-reactive protein levels. Therefore, direct evidence to depict the impacts of albumin decreases on postoperative outcomes, especially mortality, remains lacking in the present study. Fifth, the RCTs included in this systematic review and meta-analysis had rather small sample sizes. This factor definitively contributes to the significant data heterogeneity observed in this study. Moreover, high risk of bias of the included RCTs was noted. Collectively, these data will also lower the grade of evidence level of this study. Future studies with larger sample size and better quality are needed before further conclusions can be drawn.

In conclusion, preoperative protein-based oral nutrition supplements exert beneficial, but limited, effects on postoperative outcomes in geriatric patients with hip fracture undergoing surgery. Significant data heterogeneity and high risk of bias of the included studies are noted.

## Acknowledgments

This study was supported by Taipei Medical University under a TMU-CWRU pilot program grant (108-3805-018-400) and Wan Fang Hospital under an intramural fund (110-wf-eva-30), both awarded to CJ Huang. The sponsor has no role in study design; in the collection, analysis and interpretation of data; in the writing of the report; and in the decision to submit the article for publication. The authors state that they had no writing assistance.

## Author contributions

**Conceptualization:** Wen-Yi Lai, Yu-Chi Chiu, Kuo-Ching Lu, I-Tao Huang, Pei-Shan Tsai, Chun-Jen Huang.

**Data curation:** Wen-Yi Lai, Yu-Chi Chiu, Pei-Shan Tsai, Chun-Jen Huang.

**Formal analysis:** Wen-Yi Lai, Yu-Chi Chiu, Kuo-Ching Lu, I-Tao Huang, Pei-Shan Tsai, Chun-Jen Huang.

**Supervision:** Pei-Shan Tsai, Chun-Jen Huang.

**Writing – original draft:** Wen-Yi Lai, Yu-Chi Chiu, Kuo-Ching Lu, I-Tao Huang, Pei-Shan Tsai, Chun-Jen Huang.

**Conceptualization:** Wen-Yi Lai, Yu-Chi Chiu, Kuo-Ching Lu, I-Tao Huang, Pei-Shan Tsai, Chun-Jen Huang.

**Data curation:** Wen-Yi Lai, Yu-Chi Chiu, Kuo-Ching Lu, I-Tao Huang, Pei-Shan Tsai, Chun-Jen Huang.

**Formal analysis:** Wen-Yi Lai, Kuo-Ching Lu, I-Tao Huang, Pei-Shan Tsai, Chun-Jen Huang.

**Funding acquisition:** Yu-Chi Chiu, Chun-Jen Huang.

**Investigation:** Wen-Yi Lai, Yu-Chi Chiu, Kuo-Ching Lu, I-Tao Huang, Pei-Shan Tsai, Chun-Jen Huang.

**Methodology:** Wen-Yi Lai, Chun-Jen Huang.

**Project administration:** Chun-Jen Huang.

**Resources:** Yu-Chi Chiu, Chun-Jen Huang.

**Software:** Chun-Jen Huang.

**Supervision:** Yu-Chi Chiu, Pei-Shan Tsai, Chun-Jen Huang.

**Validation:** Wen-Yi Lai, Kuo-Ching Lu, I-Tao Huang, Pei-Shan Tsai, Chun-Jen Huang.

**Visualization:** Wen-Yi Lai.

**Writing – original draft:** Wen-Yi Lai, Yu-Chi Chiu, Kuo-Ching Lu, I-Tao Huang, Pei-Shan Tsai, Chun-Jen Huang.

**Writing – review & editing:** Wen-Yi Lai, Yu-Chi Chiu, Kuo-Ching Lu, I-Tao Huang, Pei-Shan Tsai, Chun-Jen Huang.
